# ﻿Three new endophytic *Apiospora* species (Apiosporaceae, Amphisphaeriales) from China

**DOI:** 10.3897/mycokeys.105.122583

**Published:** 2024-05-31

**Authors:** Xiao-Ni Yan, Chu-Long Zhang

**Affiliations:** 1 Ministry of Agriculture Key Laboratory of Molecular Biology of Crop Pathogens and Insects, Key Laboratory of Biology of Crop Pathogens and Insects of Zhejiang Province, Institute of Biotechnology, Zhejiang University, Hangzhou 310058, China Zhejiang University Hangzhou China

**Keywords:** *Apiospora*, Ascomycota, endophyte, phylogeny, taxonomy

## Abstract

*Apiospora* species are widely distributed fungi with diverse lifestyles, primarily functioning as plant pathogens, as well as exhibiting saprophytic and endophytic behaviors. This study reports the discovery of three new species of *Apiospora*, namely *A.gongcheniae*, *A.paragongcheniae*, and *A.neogongcheniae*, isolated from healthy Poaceae plants in China. These novel species were identified through a multi-gene phylogenetic analysis. The phylogenetic analysis of the combined ITS, LSU, *tef1*, and *tub2* sequence data revealed that the three new species formed a robustly supported clade with *A.garethjonesii*, *A.neogarethjonesii*, *A.setostroma*, *A.subrosea*, *A.mytilomorpha*, and *A.neobambusae*. Detailed descriptions of the newly discovered species are provided and compared with closely related species to enhance our understanding of the genus *Apiospora*.

## ﻿Introduction

*Apiospora* is an important genus of fungal Sordariomycetes, that produces a basauxic, arthrinium-like conidiogenesis (Hyde et al. 2020). The family Apiosporaceae was established to accommodate the genus *Apiospora* with the special conidiogenesis (Hyde et al. 1998). Over time, the membership of Apiosporaceae has undergone several revisions. It presently comprises several genera of fungi with similar morphology, including *Apiospora*, *Arthrinium*, *Nigrospora*, and *Neoarthrinium* (Wang et al. 2017; Pintos and Alvarado 2021; Jiang et al. 2022).

Within the family Apiosporaceae, *Apiospora* is closely related to *Arthrinium* and they were once considered as two life stages of a single taxon (Ellis 1965; Crous and Groenewald 2013; Réblová et al. 2016; Jiang et al. 2019). Morphologically, *Apiospora* and *Arthrinium* lack clear diagnostic features, although species of *Arthrinium* often produce conidia of various shapes (Minter and Cannon 2018; Pintos and Alvarado 2021), while most species of *Apiospora* have rounded lenticular conidia (Li et al. 2023; Liao et al. 2023). Ecologically, most sequenced collections of *Arthrinium* were found on Cyperaceae or Juncaceae in temperate, cold, or alpine habitats, while those of *Apiospora* were mainly collected on Poaceae, as well as various other plant host families, in a wide range of habitats, including tropical and subtropical regions (Dai et al. 2016; Jiang et al. 2018; Wang et al. 2018; Feng et al. 2021; Tian et al. 2021; Kwon et al. 2022; Monkai et al. 2022). With the addition of molecular evidence and the expansion of the sample, the latest phylogenetic analysis suggests that *Arthrinium* s. str. and *Apiospora* represent independent lineages within Apiosporaceae (Pintos and Alvarado 2021). Consequently, most species of *Arthrinium* have been reclassified under *Apiospora*. Furthermore, Pintos and Alvarado defined the exact identity of *Apiosporamontagnei* (the type species of *Apiospora*) and delineated the phylogenetic boundaries of *Apiospora* (Pintos and Alvarado 2022).

Currently, there are 176 records in *Apiospora* (Index Fungorum; http://www.indexfungorum.org/; accessed on 8 Mar 2024). These fungi primarily act as plant pathogens, causing diseases in a wide range of host plants. For example, *A.arundinis* is the causal agent for several important plant diseases, such as kernel blight of barley (Martínez-Cano et al. 1992), brown culm streak of *Phyllostachyspraecox* (Chen et al. 2014), moldy sugarcane (Liao et al. 2022), and leaf spot on *Polygonatumcyrtonema* (Gong et al. 2023). *A.marii* causes dieback of olive trees (Gerin et al. 2020), while *A.kogelbergense* leads to blight of *Bambusaintermedi* (Yin et al. 2020). Whereas, many *Apiospora* species are saprophytes, such as *A.acutiapica* (Senanayake et al. 2020), *A.garethjonesii* (Dai et al. 2016), *A.magnispora* (Zhao et al. 2023), *A.sasae* (Crous et al. 2021), and *A.thailandicum* (Dai et al. 2017). In addition, certain *Apiospora* species are reported as endophytes with wide host range, including bamboo (Wang et al. 2018), *Camelliasinensis* (Wang et al. 2018), *Wurfbainiavillosa* (Liao et al. 2023), and even hive-stored pollen (Zhao et al. 2018).

Endophytic fungi exhibit rich diversity and play a significant role in the ecosystem. In a previous study, we collected and isolated endophytic fungi from healthy Poaceae plants in China (Liu et al. 2021). In this study, three new endophytic species of *Apiospora* were identified and described based on morphological characteristics and a multi-gene phylogenetic analysis, utilizing a dataset comprising the combined nuclear ribosomal DNA internal transcribed spacer (ITS), nuclear ribosomal DNA large subunit (LSU), the translation elongation factor 1-alpha (tef1), and β-tubulin (tub2) sequences.

## ﻿Materials and methods

### ﻿Fungal isolation

In the present work, Poaceae plant samples were collected from three locations in China: Xilingol Grassland National Nature Reserve in Inner Mongolia, Xishuangbanna, Naban River Watershed National Nature Reserve in Yunnan province, and Baishanzu National Nature Reserve in Zhejiang province (Liu et al. 2021). To isolate endophytic *Apiospora* strains, healthy tissues of asymptomatic plants were first disinfected for 3 min in 75% ethanol and 10 min in 1% sodium hypochlorite, followed by three washes in sterile distilled water. The disinfected tissues were excised, and then incubated on malt extract agar (MEA) medium at 25 °C. Subsequently, the growing hyphae were transferred to potato dextrose agar (PDA) medium to obtain pure cultures.

All strains of *Apiospora* were stored in the Ministry of Agriculture Key Laboratory of Molecular Biology of Crop Pathogens and Insects, Institute of Biotechnology, Zhejiang University, Hangzhou, China. In addition, the holotype and ex-type culture were deposited in the Guangdong Microbial Culture Collection Center (GDMCC). Fungal names were registered in the Fungal Names, one of the recognised repositories of fungal taxonomy (https://nmdc.cn/fungalnames/).

### ﻿Morphological study

Morphological descriptions were recorded on PDA and MEA. The morphological characteristics of the colonies were captured with a digital camera (Canon EOS700D). The fungal structures were observed and photographed using a stereomicroscope (Leica S9D) and a Leica DM2500 microscope equipped with differential interference contrast (DIC). Measurements of conidiogenous cells and conidia were reported as follows: a-b × c-d (mean, n), where “a” and “c” represent the minimum values, “b” and “d” represent the maximum values, and the mean value and number of measurements (n) are shown in parentheses (Wang et al. 2018).

### ﻿DNA extraction, PCR amplification and sequencing

Fresh fungal mycelia from pure cultures grown on PDA at 25 °C for 5–7 d were used for DNA extraction. Genomic DNA was extracted following the method as described in Chi et al. (2009).

Polymerase chain reaction (PCR) amplification was applied to amplify four gene fragments, including ITS, LSU, *tef1*, and *tub2*. The primer pairs were used: ITS1/ITS4 for ITS (White et al. 1990), LR0R/LR5 for LSU (Rehner and Samuels 1995), EF1-728F/EF2 for *tef1* (O’Donnell et al, 1998; Carbone and Kohn 1999), and T1/Bt2b for *tub2* (Glass and Donaldson 1995; O’Donnell and Cigelnik 1997). PCR program for ITS amplification was conducted with an initial denaturation at 95 °C for 3 min, followed by 35 cycles of 95 °C for 30 s, annealing at 58 °C for 30 s, extension at 72 °C for 1 min, and a final extension at 72 °C for 7 min. The annealing temperatures were adjusted to 56 °C for LSU, *tef1*, and *tub2*.

PCR was performed using a Veriti Thermal Cycler (Waltham, MA, USA). Amplification reactions contained 10 μL of 2× Taq Plus Master Mix II (Vazyme, Nanjing, China), 0.8 μL of each primer (10 μM) (Sunya, Hangzhou, China), 0.8 μL of DNA template, and double-distilled water to reach a total volume of 20 μL. Purification and sequencing of PCR products were performed by Sunya Biotechnology Company (Hangzhou, China). All sequences generated in this study were deposited in GenBank (Table 1).

**Table 1.** Species of Apiosporaceae used in the phylogenetic analyses. Notes: Strains in this study are marked in bold. “T” indicates a type culture. NA = not available.

Species	Strain Numbers	Host and Substrates	Locality	GenBank accession numbers			
				ITS	LSU	*tef1*	*tub2*
*Apiosporaacutiapica*	KUMCC 20-0209	*Bambusabambos*	China	MT946342	MT946338	MT947359	MT947365
*Apiosporaacutiapica*	KUMCC 20-0210 ^T^	*Bambusabambos*	China	MT946343	MT946339	MT947360	MT947366
*Apiosporaadinandrae*	SAUCC 1282B-1 ^T^	Diseased leaves of *Adinandraglischroloma*	China	OR739431	OR739572	OR753448	OR757128
*Apiosporaadinandrae*	SAUCC 1282B-2	Diseased leaves of *Adinandraglischroloma*	China	OR739432	OR739573	OR753449	OR757129
*Apiosporaagari*	KUC21333, SFC20161014-M18 ^T^	*Agarumcribrosum*	South Korea	MH498520	MH498440	MH544663	MH498478
*Apiosporaaquatic*	MFLU 18-1628, S-642 ^T^	Submerged wood	China	MK828608	MK835806	NA	NA
*Apiosporaarctoscopi*	KUC21331, SFC20200506-M05 ^T^	Eggs of *Arctoscopusjaponicus*	South Korea	MH498529	MH498449	MN868918	MH498487
*Apiosporaarundinis*	CBS 124788	Living leaves of *Fagussylvatica*	Switzerland	KF144885	KF144929	KF145017	KF144975
*Apiosporaarundinis*	LC4951	*Dichotomanthestristaniicarpa*	China	KY494698	KY494774	KY705097	KY705168
*Apiosporaaseptata*	KUNCC 23-14169 ^T^	Living roots of *Dicranopterispedata*	China	OR590341	OR590335	OR634949	OR634943
*Apiosporaaurea*	CBS 244.83 ^T^	Air	Spain	AB220251	KF144935	KF145023	KF144981
*Apiosporabalearica*	CBS 145129, AP24118 ^T^	Poaceae plant	Spain	MK014869	MK014836	MK017946	MK017975
*Apiosporabambusicola*	MFLUCC 20-0144 ^T^	*Schizostachyumbrachycladum*	Thailand	MW173030	MW173087	MW183262	NA
*Apiosporabawanglingensis*	SAUCC BW0444 ^T^	Leaves of *Indocalamuslongiauritus*	China	OR739429	OR739570	OR753446	OR757126
*Apiosporabiserialis*	CGMCC 3.20135 ^T^	Bamboo	China	MW481708	MW478885	MW522938	MW522955
*Apiosporacamelliae-sinensis*	CGMCC 3.18333, LC5007 ^T^	*Camelliasinensis*	China	KY494704	KY494780	KY705103	KY705173
*Apiosporacamelliae-sinensis*	LC8181	*Brassicarapa*	China	KY494761	KY494837	KY705157	KY705229
*Apiosporacannae*	ZHKUCC 22-0139	Leaves of *Canna* sp.	China	OR164902	OR164949	OR166286	OR166322
*Apiosporacannae*	ZHKUCC 22-0127 ^T^	Leaves of *Canna* sp.	China	OR164901	OR164948	OR166285	OR166321
*Apiosporachiangraiense*	MFLUCC 21-0053 ^T^	Dead culms of bamboo	Thailand	MZ542520	MZ542524	NA	MZ546409
*Apiosporachromolaenae*	MFLUCC 17-1505 ^T^	*Chromolaenaodorata*	Thailand	MT214342	MT214436	MT235802	NA
*Apiosporacordylinae*	GUCC 10026	*Cordylinefruticosa*	China	MT040105	NA	MT040126	MT040147
*Apiosporacordylinae*	GUCC 10027 ^T^	*Cordylinefruticosa*	China	MT040106	NA	MT040127	MT040148
*Apiosporacoryli*	CFCC 58978 ^T^	Dead plant culms of *Corylusyunnanensis*	China	OR125564	OR133586	OR139974	OR139978
*Apiosporacoryli*	CFCC 58979 ^T^	Dead plant culms of *Corylusyunnanensis*	China	OR125565	OR133587	OR139975	OR139979
*Apiosporacyclobalanopsidis*	CGMCC 3.20136 ^T^	*Cyclobalanopsidisglauca*	China	MW481713	MW478892	MW522945	MW522962
*Apiosporacyclobalanopsidis*	GZCC 20-0103	*Cyclobalanopsidisglauca*	China	MW481714	MW478893	MW522946	MW522963
*Apiosporadendrobii*	MFLUCC 14-0152 ^T^	Roots of *Dendrobiumharveyanum*	Thailand	MZ463151	MZ463192	NA	NA
*Apiosporadematiacea*	KUNCC 23-14202 ^T^	Living stems of *Dicranopterisampla*	China	OR590346	OR590339	OR634953	OR634948
*Apiosporadescalsii*	CBS 145130 ^T^	*Ampelodesmosmauritanicus*	Spain	MK014870	MK014837	MK017947	MK017976
*Apiosporadichotomanthi*	CGMCC 3.18332, LC4950 ^T^	*Dichotomanthestristaniicarpa*	China	KY494697	KY494773	KY705096	KY705167
*Apiosporadichotomanthi*	LC8175	*Dichotomanthestristaniicarpa*	China	KY494755	KY494831	KY705151	KY705223
*Apiosporadicranopteridis*	KUNCC23-14171 ^T^	Living stems of *Dicranopterispedata*	China	OR590342	OR590336	OR634950	OR634944
*Apiosporadicranopteridis*	KUNCC23-14177	Roots of *Dicranopterispedata*	China	OR590343	OR590337	OR634951	OR634945
*Apiosporadongyingensis*	SAUCC 0302 ^T^	Leaves of bamboo	China	OP563375	OP572424	OP573264	OP573270
*Apiosporadongyingensis*	SAUCC 0303	Leaves of bamboo	China	OP563374	OP572423	OP573263	OP573269
*Apiosporaelliptica*	ZHKUCC 22-0131 ^T^	Dead stems of unknown plant	China	OR164905	OR164952	OR166284	OR166323
*Apiosporaelliptica*	ZHKUCC 22-0140	Dead stems of unknown plant	China	OR164906	OR164953	NA	OR166324
*Apiosporaendophytica*	ZHKUCC 23-0006 ^T^	Living leaves of *Wurfbainiavillosa*	China	OQ587996	OQ587984	OQ586062	OQ586075
*Apiosporaendophytica*	ZHKUCC 23-0007	Living leaves of *Wurfbainiavillosa*	China	OQ587997	OQ587985	OQ586063	OQ586076
*Apiosporaesporlensis*	CBS 145136 ^T^	*Phyllostachysaurea*	Spain	MK014878	MK014845	MK017954	MK017983
*Apiosporaesporlensis*	UNIPAMPA010	Living leaves of the Antarctic Hairgrass *Deschampsiaantarctica*	Antarctica	MN947641	genome	genome	genome
*Apiosporaeuphorbiae*	IMI 285638b	*Bambusa* sp.	Bangladesh	AB220241	AB220335	NA	AB220288
*Apiosporafermenti*	KUC21288, SFC20140423-M86	Seaweeds	South Korea	MF615230	NA	MH544668	MF615235
*Apiosporafermenti*	KUC21289 ^T^	Seaweeds	South Korea	MF615226	MF615213	MH544667	MF615231
*Apiosporagaoyouensis*	CFCC 52301^T^	*Phragmitesaustralis*	China	MH197124	NA	MH236793	MH236789
*Apiosporagaoyouensis*	CFCC 52302	*Phragmitesaustralis*	China	MH197125	NA	MH236794	MH236790
*Apiosporagarethjonesii*	GZCC 20-0115	Dead culms of bamboo	China	MW481715	MW478894	MW522947	NA
*Apiosporagarethjonesii*	KUMCC 16-0202, JHB004, HKAS 96289 ^T^	Dead culms of bamboo	China	KY356086	KY356091	NA	NA
*Apiosporagarethjonesii*	SICAUCC 22-0027	Bamboo	China	ON228603	ON228659	NA	ON237651
*Apiosporagarethjonesii*	SICAUCC 22-0028	Bamboo	China	ON228606	ON228662	NA	ON237654
*Apiosporagelatinosa*	GZAAS 20-0107	Bamboo	China	MW481707	MW478889	MW522942	MW522959
*Apiosporagelatinosa*	HKAS 11962 ^T^	Bamboo	China	MW481706	MW478888	MW522941	MW522958
*Apiosporaglobosa*	KUNCC 23-14210 ^T^	Living stems of *Dicranopterislinearis*	China	OR590347	OR590340	OR634954	NA
***Apiosporagongcheniae***	**GDMCC 3.1045, YNE00465 ^T^**	**Living stems of Oryzameyerianasubsp.granulata**	**China**	**PP033259**	**PP033102**	**PP034683**	**PP034691**
***Apiosporagongcheniae***	**YNE00565**	**Living stems of Oryzameyerianasubsp.granulata**	**China**	**PP033260**	**PP033103**	**PP034684**	**PP034692**
*Apiosporaguangdongensis*	ZHKUCC 23-0004 ^T^	Living leaves of *Wurfbainiavillosa*	China	OQ587994	OQ587982	OQ586060	OQ586073
*Apiosporaguangdongensis*	ZHKUCC 23-0005	Living leaves of *Wurfbainiavillosa*	China	OQ587995	OQ587983	OQ586061	OQ586074
*Apiosporaguiyangensis*	HKAS 102403 ^T^	Dead culms of Poaceae	China	MW240647	MW240577	MW759535	MW775604
*Apiosporaguiyangensis*	KUNCC 22-12539	Poaceae plant	China	OQ029540	OQ029613	OQ186444	OQ186446
*Apiosporaguizhouensis*	CGMCC 3.18334, LC5322 ^T^	Air in karst cave	China	KY494709	KY494785	KY705108	KY705178
*Apiosporaguizhouensis*	LC5318	Air in karst cave	China	KY494708	KY494784	KY705107	KY705177
*Apiosporahainanensis*	SAUCC 1681 ^T^	Leaves of bamboo	China	OP563373	OP572422	OP573262	OP573268
*Apiosporahainanensis*	SAUCC 1682	Leaves of bamboo	China	OP563372	OP572421	OP573261	OP573267
*Apiosporahispanica*	IMI 326877 ^T^	Beach sands	Spain	AB220242	AB220336	NA	AB220289
*Apiosporahydei*	CBS 114990 ^T^	Culms of *Bambusatuldoides*	China	KF144890	KF144936	KF145024	KF144982
*Apiosporahydei*	LC7103	Leaves of bamboo	China	KY494715	KY494791	KY705114	KY705183
*Apiosporahyphopodii*	JHB003, HKAS 96288	Bamboo	China	KY356088	KY356093	NA	NA
*Apiosporahyphopodii*	MFLUCC 15-003 ^T^	*Bambusatuldoides*	Thailand	KR069110	NA	NA	NA
*Apiosporahyphopodii*	SICAUCC 22-0034	Bamboo	China	ON228605	ON228661	NA	ON237653
*Apiosporahysterina*	AP12118	*Phyllostachysaurea*	Spain	MK014877	KM014844	MK017953	MK017982
*Apiosporahysterina*	AP29717	*Phyllostachysaurea*	Spain	MK014875	MK014842	MK017952	MK017981
*Apiosporahysterina*	ICPM 6889 ^T^	Bamboo	New Zealand	MK014874	MK014841	MK017951	MK017980
*Apiosporaiberica*	CBS 145137, AP10118 ^T^	*Arundodonax*	Portugal	MK014879	MK014846	MK017955	MK017984
*Apiosporaintestine*	CBS 135835	Gut of grasshopper	India	KR011352	MH877577	KR011351	KR011350
*Apiosporaintestine*	MFLUCC 21-0052 ^T^	Dead culms of bamboo	Thailand	MZ542521	MZ542525	MZ546406	MZ546410
*Apiosporaitalic*	CBS 145138, AP221017 ^T^	*Arundodonax*	Italy	MK014880	MK014847	MK017956	MK017985
*Apiosporaitalic*	CBS 145139	*Phragmitesaustralis*	Spain	MK014881	MK014848	NA	MK017986
*Apiosporajatrophae*	CBS 134262, MMI00052 ^T^	Living *Jatrophapodagrica*	India	JQ246355	NA	NA	NA
*Apiosporajiangxiensis*	CGMCC 3.18381, LC4577 ^T^	*Maesa* sp.	China	KY494693	KY494769	KY705092	KY705163
*Apiosporajiangxiensis*	LC4578	*Camelliasinensis*	China	KY494694	KY494770	KY705093	KY705164
*Apiosporakogelbergensis*	CBS 113332	*Cannomoisvirgata*	South Africa	KF144891	KF144937	KF145025	KF144983
*Apiosporakogelbergensis*	CBS 113333 ^T^	Dead culms of Restionaceae	South Africa	KF144892	KF144938	KF145026	KF144984
*Apiosporakoreanum*	KUC21332, SFC20200506-M06 ^T^	Eggs of *Arctoscopusjaponicus*	South Korea	MH498524	MH498444	MH544664	MH498482
*Apiosporakoreanum*	KUC21348	Eggs of *Arctoscopusjaponicus*	South Korea	MH498523	NA	MN868927	MH498481
*Apiosporalageniformis*	KUC21686 ^T^	Culms of *Phyllostachysnigra*	Korea	ON764022	ON787761	ON806626	ON806636
*Apiosporalageniformis*	KUC21687	Culms of *Phyllostachysnigra*	Korea	ON764023	ON787764	ON806627	ON806637
*Apiosporalocuta-pollinis*	LC11683 ^T^	*Brassicacampestris*	China	MF939595	NA	MF939616	MF939622
*Apiosporalongistroma*	MFLUCC 11-0479	Dead culms of bamboo	Thailand	KU940142	KU863130	NA	NA
*Apiosporalongistroma*	MFLUCC11-0481 ^T^	Dead culms of bamboo	Thailand	KU940141	KU863129	NA	NA
*Apiosporalophatheri*	CFCC 58975 ^T^	Diseased leaves of *Lophatherumgracile*	China	OR125566	OR133588	OR139970	OR139980
*Apiosporalophatheri*	CFCC 58976 ^T^	Diseased leaves of *Lophatherumgracile*	China	OR125567	OR133589	OR139971	OR139981
*Apiosporamachili*	SAUCC 1175A-4 ^T^	Diseased leaves of *Machilusnanmu* of Machilusnanmu	China	OR739433	OR739574	OR753450	OR757130
*Apiosporamachili*	SAUCC 1175	Diseased leaves of *Machilusnanmu* of Machilusnanmu	China	OQ592560	OQ615289	OQ613333	OQ613307
*Apiosporamagnispora*	ZHKUCC 22-0001 ^T^	Dead stems of *Bambusatextilis*	China	OM728647	OM486971	OM543543	OM543544
*Apiosporamalaysiana*	CBS 102053 ^T^	*Macarangahullettii*	Malaysia	KF144896	KF144942	KF145030	KF144988
*Apiosporamarianiae*	AP18219 ^T^	Dead stems of *Phleumpratense*	Spain	ON692406	ON692422	ON677180	ON677186
*Apiosporamarii*	CBS 497.90 ^T^	Beach sands	Spain	AB220252	KF144947	KF145035	KF144993
*Apiosporamarinum*	KUC21328, SFC20140423-M02 ^T^	Seaweeds	South Korea	MH498538	MH498458	MH544669	MH498496
*Apiosporamediterranea*	IMI 326875 ^T^	Air	Spain	AB220243	AB220337	NA	AB220290
*Apiosporaminutispora*	1.70E-042 ^T^	Mountain soils	South Korea	LC517882	NA	LC518889	LC518888
*Apiosporamontagnei*	AP19421	*Arundomicrantha*	Spain	ON692418	ON692425	ON677183	ON677189
*Apiosporamontagnei*	AP301120, CBS 148707, PC:0125164 ^T^	*Arundomicrantha*	Spain	ON692408	ON692424	ON677182	ON677188
*Apiosporamori*	MFLUCC 20-0181 ^T^	Dead leaves of *Morusaustralis*	China	MW114313	MW114393	NA	NA
*Apiosporamori*	NCYUCC 19-0340	Dead leaves of *Morusaustralis*	China	MW114314	MW114394	NA	NA
*Apiosporamukdahanensis*	MFLUCC 22-0056 ^T^	Dead leaves of bamboo	Thailand	OP377735	OP377742	NA	NA
*Apiosporamultiloculata*	MFLUCC 21-0023 ^T^	Dead culms of Bambusae	Thailand	OL873137	OL873138	NA	OL874718
*Apiosporamytilomorpha*	DAOM 214595 ^T^	Dead blades of *Andropogon* sp.	India	KY494685	NA	NA	NA
*Apiosporaneobambusae*	CGMCC 3.18335, LC7106 ^T^	Leaves of bamboo	China	KY494718	KY494794	KY806204	KY705186
*Apiosporaneobambusae*	LC7107	Leaves of bamboo	China	KY494719	KY494795	KY705117	KY705187
*Apiosporaneobambusae*	LC7124	Leaves of bamboo	China	KY494727	KY494803	KY806206	KY705195
*Apiosporaneochinensis*	CFCC 53036 ^T^	*Fargesiaqinlingensis*	China	MK819291	NA	MK818545	MK818547
*Apiosporaneochinensis*	CFCC 53037	*Fargesiaqinlingensis*	China	MK819292	NA	MK818546	MK818548
*Apiosporaneogarethjonesii*	KUMCC 18-0192, HKAS 102408 ^T^	Dead culms of Bambusae	China	MK070897	MK070898	NA	NA
***Apiosporaneogongcheniae***	**GDMCC 3.1047, YNE01248 ^T^**	**Living stems of Poaceae plant**	**China**	**PP033263**	**PP033106**	**PP034687**	**PP034695**
***Apiosporaneogongcheniae***	**YNE01260**	**Living stems of Poaceae plant**	**China**	**PP033264**	**PP033107**	**PP034688**	**PP034696**
*Apiosporaneosubglobosa*	JHB 006	Bamboo	China	KY356089	KY356094	NA	NA
*Apiosporaneosubglobosa*	JHB 007 ^T^	Bamboo	China	KY356090	KY356095	NA	NA
*Apiosporaobovata*	CGMCC 3.18331, LC4940 ^T^	*Lithocarpus* sp.	China	KY494696	KY494772	KY705095	KY705166
*Apiosporaobovata*	LC8177	*Lithocarpus* sp.	China	KY494757	KY494833	KY705153	KY705225
*Apiosporaoenotherae*	CFCC 58972	Diseased leaves of *Oenotherabiennis*	China	OR125568	OR133590	OR139972	OR139982
*Apiosporaoenotherae*	LS 395	Diseased leaves of *Oenotherabiennis*	China	OR125569	OR133591	OR139973	OR139983
*Apiosporaovate*	CBS 115042 ^T^	*Arundinariahindsii*	China	KF144903	KF144950	KF145037	KF144995
*Apiosporapallidesporae*	ZHKUCC 22-0129 ^T^	Dead wood of unknown host	China	OR164903	OR164950	NA	NA
*Apiosporapallidesporae*	ZHKUCC 22-0142	Dead wood of unknown host	China	OR164904	OR164951	NA	NA
***Apiosporaparagongcheniae***	**GDMCC 3.1046, YNE00992 ^T^**	**Living stems of Poaceae plant**	**China**	**PP033261**	**PP033104**	**PP034685**	**PP034693**
***Apiosporaparagongcheniae***	**YNE01259**	**Living stems of Poaceae plant**	**China**	**PP033262**	**PP033105**	**PP034686**	**PP034694**
*Apiosporaparaphaeosperma*	MFLUCC 13-0644 ^T^	Dead culms of bamboo	Thailand	KX822128	KX822124	NA	NA
*Apiosporaparaphaeosperma*	KUC21488	Culms of bamboo	Korea	ON764024	ON787763	ON806628	ON806638
*Apiosporaphragmitis*	CPC 18900 ^T^	*Phragmitesaustralis*	Italy	KF144909	KF144956	KF145043	KF145001
*Apiosporaphyllostachydis*	MFLUCC 18-1101 ^T^	*Phyllostachysheteroclada*	China	MK351842	MH368077	MK340918	MK291949
*Apiosporapiptatheri*	CBS 145149, AP4817A ^T^	*Piptatherummiliaceum*	Spain	MK014893	MK014860	MK017969	NA
*Apiosporapiptatheri*	SAUCC BW0455	Diseased leaves of *Indocalamuslongiauritus*	China	OR739430	OR739571	OR753447	OR757127
*Apiosporapseudomarii*	GUCC 10228 ^T^	Leaves of *Aristolochiadebilis*	China	MT040124	NA	MT040145	MT040166
*Apiosporapseudohyphopodii*	KUC21680 ^T^	Culms of *Phyllostachyspubescens*	Korea	ON764026	ON787765	ON806630	ON806640
*Apiosporapseudohyphopodii*	KUC21684	Culms of *Phyllostachyspubescens*	Korea	ON764027	ON787766	ON806631	ON806641
*Apiosporapseudoparenchymatica*	CGMCC 3.18336, LC7234 ^T^	Leaves of bamboo	China	KY494743	KY494819	KY705139	KY705211
*Apiosporapseudoparenchymatica*	LC8173	Leaves of bamboo	China	KY494753	KY494829	KY705149	KY705221
*Apiosporapseudorasikravindrae*	KUMCC 20-0208 ^T^	*Bambusadolichoclada*	China	MT946344	NA	MT947361	MT947367
*Apiosporapseudosinensis*	CPC 21546 ^T^	Leaves of bamboo	Netherlands	KF144910	KF144957	KF145044	MN868936
*Apiosporapseudosinensis*	SAUCC 0221	Leaves of bamboo	China	OP563377	OP572426	OP573266	OP573272
*Apiosporapseudospegazzinii*	CBS 102052 ^T^	*Macarangahullettii*	Malaysia	KF144911	KF144958	KF145045	KF145002
*Apiosporapterosperma*	CBS 123185	*Machaerinasinclairii*	New Zealand	KF144912	KF144959	NA	KF145003
*Apiosporapterosperma*	CPC 20193, CBS 134000 ^T^	*Lepidospermagladiatum*	Australia	KF144913	KF144960	KF145046	KF145004
*Apiosporapusillispermum*	KUC21321 ^T^	Seaweeds	South Korea	MH498533	MH498453	MN868930	MH498491
*Apiosporapusillispermum*	KUC21357	Seaweeds	South Korea	MH498532	NA	MN868931	MH498490
*Apiosporaqinlingensis*	CFCC 52303 ^T^	*Fargesiaqinlingensis*	China	MH197120	NA	MH236795	MH236791
*Apiosporaqinlingensis*	CFCC 52304	*Fargesiaqinlingensis*	China	MH197121	NA	MH236796	MH236792
*Apiosporarasikravindrae*	LC8179	*Brassicarapa*	China	KY494759	KY494835	KY705155	KY705227
*Apiosporarasikravindrae*	MFLUCC 21-0051	Dead culms of bamboo	Thailand	MZ542523	MZ542527	MZ546408	MZ546412
*Apiosporasacchari*	CBS 372.67	Air	Not mentioned	KF144918	KF144964	KF145049	KF145007
*Apiosporasacchari*	CBS 664.74	Soils under *Callunavulgaris*	Netherlands	KF144919	KF144965	KF145050	KF145008
*Apiosporasaccharicola*	CBS 191.73	Air	Netherlands	KF144920	KF144966	KF145051	KF145009
*Apiosporasaccharicola*	CBS 831.71	Not mentioned	Netherlands	KF144922	KF144969	KF145054	KF145012
*Apiosporasargassi*	KUC21228 ^T^	*Sargassumfulvellum*	South Korea	KT207746	KT207696	MH544677	KT207644
*Apiosporasargassi*	KUC21232	Seaweeds	South Korea	KT207750	NA	MH544676	KT207648
*Apiosporasasae*	CPC 38165, CBS 146808 ^T^	Dead culms of *Sasaveitchii*	Netherlands	MW883402	MW883797	MW890104	MW890120
*Apiosporaseptata*	CGMCC 3.20134, CS19-8 ^T^	Bamboo	China	MW481711	MW478890	MW522943	MW522960
*Apiosporaseptata*	GZCC 20-0109	Bamboo Food	China	MW481712	MW478891	MW522944	MW522961
*Apiosporaserenensis*	IMI 326869 ^T^	Excipients, atmosphere and home dust	Spain	AB220250	AB220344	NA	AB220297
*Apiosporasetariae*	CFCC 54041 ^T^	Decaying culms of *Setariaviridis*	China	MT492004	NA	MW118456	MT497466
*Apiosporasetariae*	MT492005	*Setariaviridis*	China	MT492005	NA	MW118457	MT497467
*Apiosporasetostroma*	KUMCC 19-0217	Dead branches of bamboo	China	MN528012	MN528011	MN527357	NA
*Apiosporasichuanensis*	HKAS 107008 ^T^	Dead culms of Poaceae	China	MW240648	MW240578	MW759536	MW775605
*Apiosporasorghi*	URM 93000, URM 7417 ^T^	*Sorghumbicolor*	Brazil	MK371706	NA	NA	MK348526
*Apiosporasphaerosperma*	CBS 114314	Leaves of *Hordeumvulgare*	Iran	KF144904	KF144951	KF145038	KF144996
*Apiosporasphaerosperma*	CBS 114315	Leaves of *Hordeumvulgare*	Iran	KF144905	KF144952	KF145039	KF144997
*Apiosporastipae*	CPC 38101, CBS 146804 ^T^	Dead culms of *Stipagigantea*	Spain	MW883403	MW883798	MW890082	MW890121
*Apiosporasubglobosa*	MFLUCC 11-0397 ^T^	Dead culms of bamboo	Thailand	KR069112	KR069113	NA	NA
*Apiosporasubrosea*	CGMCC 3.18337, LC7292 ^T^	Leaves of bamboo	China	KY494752	KY494828	KY705148	KY705220
*Apiosporasubrosea*	LC7291	Leaves of bamboo	China	KY494751	KY494827	KY705147	KY705219
*Apiosporataeanense*	KUC21322^T^	Seaweeds	South Korea	MH498515	NA	MH544662	MH498473
*Apiosporataeanense*	KUC21359	Seaweeds	South Korea	MH498513	NA	MN868935	MH498471
*Apiosporathailandica*	MFLUCC 15-0199	Dead culms of bamboo	Thailand	KU940146	KU863134	NA	NA
*Apiosporathailandica*	MFLUCC 15-0202 ^T^	Dead culms of bamboo	Thailand	KU940145	KU863133	NA	NA
*Apiosporatropica*	MFLUCC 21-0056	Dead culms of Bambusoideae	Thailand	OK491657	OK491653	NA	OK560922
*Apiosporawurfbainiae*	ZHKUCC 23-0008 ^T^	*Wurfbainiavillosa*	China	OQ587998	OQ587986	OQ586064	OQ586077
*Apiosporawurfbainiae*	ZHKUCC 23-0009	*Wurfbainiavillosa*	China	OQ587999	OQ587987	OQ586065	OQ586078
*Apiosporavietnamensis*	IMI 99670 ^T^	*Citrussinensis*	Vietnam	KX986096	KX986111	NA	KY019466
*Apiosporaxenocordella*	CBS 478.86 ^T^	Soils from roadway	Zimbabwe	KF144925	KF144970	KF145055	KF145013
*Apiosporaxenocordella*	CBS 595.66	Soils	Austria	KF144926	KF144971	NA	NA
*Apiosporaxishuangbannaensis*	KUMCC 21-0695 ^T^	*Rhinolophuspusillus*	China	ON426832	OP363248	OR025969	OR025930
*Apiosporaxishuangbannaensis*	KUMCC 21-0696	*Rhinolophuspusillus*	China	ON426833	OP363249	OR025970	OR025931
*Apiosporayunnana*	DDQ 00281	*Phyllostachysnigra*	China	KU940148	KU863136	NA	NA
*Apiosporayunnana*	MFLUCC 15-1002 ^T^	*Phyllostachysnigra*	China	KU940147	KU863135	NA	NA
*Apiosporayunnanensis*	ZHKUCC 23-0014 ^T^	Dead stems of grass	China	OQ588004	OQ587992	OQ586070	OQ586083
*Apiosporayunnanensis*	ZHKUCC 23-0015	Dead stems of grass	China	OQ588005	OQ587993	OQ586071	OQ586084
*Arthriniumaustriacum*	GZU 345004	*Carexpendula*	Austria	MW208928	NA	NA	NA
*Arthriniumaustriacum*	GZU 345006	*Carexpendula*	Austria	MW208929	MW208860	NA	NA
*Arthriniumcaricicola*	CBS 145127, AP23518	*Carexericetorum*	China	MK014871	MK014838	MK017948	MK017977
*Arthriniumcaricicola*	CBS 145903, CPC33297 ^T^	Dead and attached leaves	Germany	MN313782	MN317266	NA	MN313861
*Arthriniumcrenatum*	AG19066, CBS 146353 ^T^	*Carex* sp.	France	MW208931	MW208861	MW221917	MW221923
*Arthriniumcurvatum*	AP25418	Leaves of *Carex* sp.	China	MK014872	MK014839	MK017949	NA
*Arthriniumjaponicum*	IFO 30500	*Carexdespalata*	Japan	AB220262	AB220356	NA	AB220309
*Arthriniumjaponicum*	IFO 31098	Leaves of *Carexdespalata*	Japan	AB220264	AB220358	NA	AB220311
*Arthriniumluzulae*	AP7619-3	*Luzulasylvatica*	Spain	MW208937	MW208863	MW221919	MW221925
*Arthriniummorthieri*	GZU 345043	*Cyperaceaecarex*	Austria	MW208938	MW208864	MW221920	MW221926
*Arthriniumphaeospermum*	AP25619, CBS 146355	Poaceae plant	Norway	MW208943	MW208865	NA	NA
*Arthriniumpuccinioides*	CBS 549.86	*Lepidospermagladiatum*	Germany	AB220253	AB220347	NA	AB220300
*Arthriniumsporophleoides*	GZU 345102	*Carex ﬁrma*	Austria	MW208944	MW208866	NA	MW221927
*Arthriniumsporophleum*	AP21118, CBS 145154	Dead leaves of *Juncus* sp.	Spain	MK014898	MK014865	MK017973	MK018001
*Nigrosporaguilinensis*	CGMCC 3.18124, LC 3481 ^T^	*Camelliasinensis*	China	KX985983	KX986113	KY019292	KY019459
*Nigrosporaguilinensis*	LC 7301	Stems of *Nelumbo* sp.	China	KX986063	NA	KY019404	KY019608
*Nigrosporahainanensis*	CGMCC 3.18129, LC 7030 ^T^	Leaves of *Musaparadisiaca*	China	KX986091	KX986112	KY019415	KY019464
*Nigrosporahainanensis*	LC 6979	Leaves of *Musaparadisiaca*	China	KX986079	NA	KY019416	KY019586
*Nigrosporapyriformis*	CGMCC 3.18122, LC 2045 ^T^	*Citrussinensis*	China	KX985940	KX986100	KY019290	KY019457
*Nigrosporapyriformis*	LC 2688	*Linderaaggregata*	China	KX985941	NA	KY019297	KY019468
*Nigrosporavesicularis*	CGMCC 3.18128, LC 7010 ^T^	Leaves of *Musaparadisiaca*	China	KX986088	KX986099	KY019294	KY019463
*Nigrosporavesicularis*	LC 0322	Unknown host plant	Thailand	KX985939	NA	KY019296	KY019467
*Neoarthriniumlithocarpicola*	CFCC 54456 ^T^	*Lithocarpusglaber*	China	ON427580	ON427582	NA	ON456914
*Neoarthriniumlithocarpicola*	CFCC 55883	*Lithocarpusglaber*	China	ON427581	ON427583	NA	ON456915
*Neoarthriniumtrachycarpi*	CFCC 53038	*Trachycarpusfortune*	China	MK301098	NA	MK303396	MK303394
*Neoarthriniumtrachycarpi*	CFCC 53039	*Trachycarpusfortune*	China	MK301099	NA	MK303397	MK303395
*Sporocadustrimorphus*	CFCC 55171	Rose	China	OK655798	OK560389	OL814555	OM401677
*Sporocadustrimorphus*	ROC 113	Rose	China	OK655799	OK560390	OL814556	OM401678

### ﻿Phylogenetic analyses

The quality of obtained sequences was assessed using Chromas v.2.6.6 and the sequences were assembled using SeqMan v.7.1.0. The reference sequences were retrieved from GenBank. All sequences, including the reference sequences, were aligned in batches with MAFFT (Katoh and Standley 2013), manually correcting the resulting alignment by MEGA v.11.0.13 where necessary. A single alignment was made using ITS, LSU, *tef1* region including partial exon 4 and partial exon 5 (the largest exon), *tub2* region including exon 2, exon 3, and partial exon 4. Then phylogenetic analyses were conducted using partial sequences of the above four loci. The sequences were trimmed and concatenated, and subsequent phylogenetic analyses were performed in PhyloSuite platform (Zhang et al. 2020). ModelFinder (Kalyaanamoorthy et al. 2017) was used to select the best-fit partition model (Edge-unlinked) using the BIC criterion. Maximum likelihood (ML) phylogenies were inferred using IQ-TREE (Nguyen et al. 2015) under Edge-linked partition model for 5000 ultrafast (Minh et al. 2013) bootstraps. Bayesian Inference (BI) phylogenies were inferred using MrBayes 3.2.6 (Ronquist et al. 2012) under partition model, in which the initial 27% of sampled data were discarded as burn-in. The resulting phylogenetic tree was visualized in FigTree v1.4.3. (http:/tree.bio.ed.ac.uk/software/figtree/) with maximum likelihood bootstrap proportions (MLBP) greater than 70% and Bayesian inference posterior probabilities (BIPP) greater than 0.90, as shown at the nodes. The phylogram was edited in Adobe Illustrator v.27.5 (Adobe Systems Inc., USA). All GenBank accession numbers of sequences used in this study are provided in Table 1.

## ﻿Results

### ﻿Phylogeny

**Figure 1.**
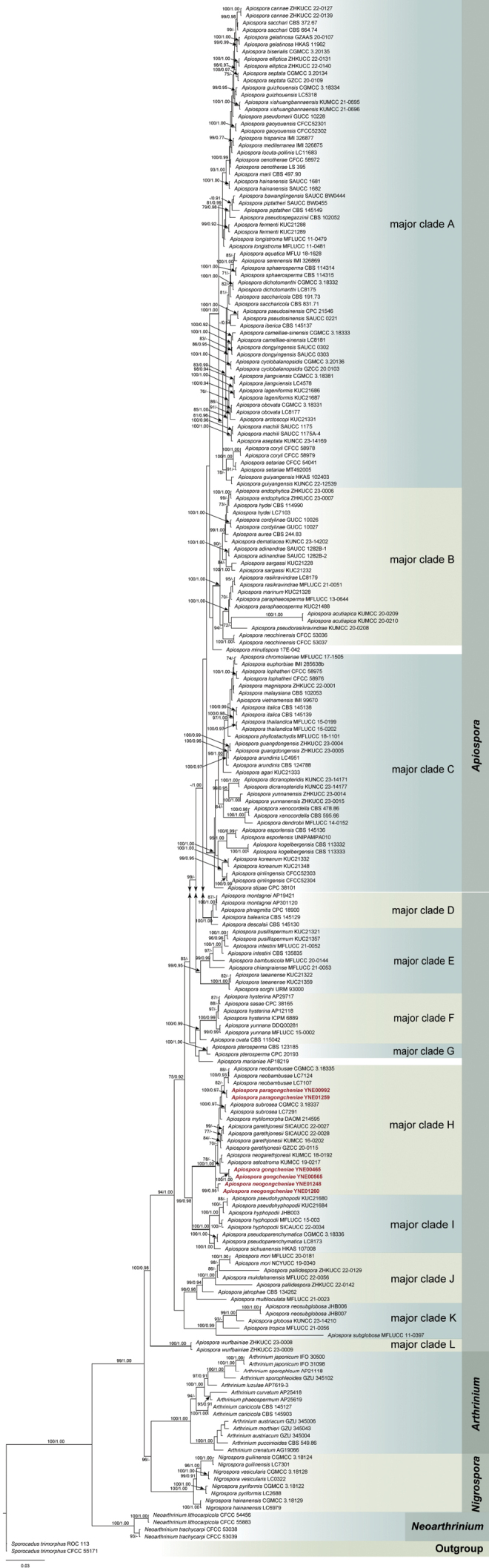
Phylogenetic tree of *Apiospora* based on the combined ITS, LSU, *tef1*, and *tub2* sequences alignment. Maximum likelihood bootstrap proportions ≥70% (left) and Bayesian inference posterior probability ≥0.90 (right) are indicated at nodes (MLBP/BIPP). *Sporocadustrimorphus* (CFCC 55171 and ROC 113) are chosen as the outgroup taxa. The novel species from this study are highlighted in red.

The combined ITS, LSU, *tef1*, and *tub2* dataset encompassed 215 strains, including six newly sequenced strains, with *Sporocadustrimorphus* CFCC 55171 and ROC 113 serving as the outgroup taxa, and representative species of *Arthrinium*, *Nigrospora*, and *Neoarthrinium* as the sister groups. The multi-locus sequence dataset comprised 2,081 characters, including gaps, with the following character ranges: ITS (1-352), LSU (353-1149), *tef1* (1150-1775), and *tub2* (1776-2081). The topologies of phylogenetic trees generated by ML and BI analyses were congruent, and the BI tree with MLBP and BIPP is presented in Fig. 1.

The phylogenetic analysis revealed that the species of *Apiospora*, *Arthrinium*, *Nigrospora*, and *Neoarthrinium* formed four well-supported distinct lineages. Within the genus *Apiospora*, the 187 strains, encompassing six newly sequenced strains, formed twelve well-supported major clades. The six endophytic strains clustered within one of the major clades H, along with *A.garethjonesii*, *A.neogarethjonesii*, *A.setostroma*, *A.subrosea*, *A.mytilomorpha*, and *A.neobambusae*. Concurrently, the six endophytic strains segregated into three independent clades with robust supported values, indicating the presence of three novel species. These novel taxa are formally described herein and assigned the new names *A.gongcheniae*, *A.paragongcheniae*, and *A.neogongcheniae*.

### ﻿Taxonomy

#### ﻿ Apiospora gongcheniae

Taxon classificationFungiXylarialesApiosporaceae

C. L. Zhang sp. nov.

3CB66A0C-DA1A-52F5-B42B-C85EE95BDB90

Fungal Names: FN 571885

Fig. 2

##### Etymology.

Named after Prof. Gongchen Wang in recognition of her significant contribution to the fields of mycology and plant pathology in China.

##### Type.

China, Yunnan Province: Xishuangbanna, Naban River Watershed National Nature Reserve, 22°04'N, 100°32'E, on the stems of Oryzameyerianasubsp.granulata, Aug 2015, J.J. Chen, YNE00465 (holotype GDMCC 3.1045, stored in a metabolically inactive state); ex-type culture YNE00465.

##### Description.

***Asexual morph***: Hyphae hyaline, branched, septate, smooth, 1.1–2.6 μm diameter (mean = 1.7 μm, n = 30). Conidiophores reduced to conidiogenous cells. Conidiogenous cells hyaline to pale brown, erect, verrucose, cylindrical with tiny denticles, clustered in groups, sometimes aggregated in clusters on hyphae or sporodochia, 3.5–9.4 × 1.9–5.2 μm (mean = 5.6 × 3.1 μm, n = 30). Conidia pale brown to dark brown, smooth, granular, globose to subglobose in surface view, lenticular to side view with a pale longitudinal germ slit, with obvious central basal scar, 8.0–17.0 × 6.8–16.1 μm (mean = 13.6 × 11.6 μm, n = 50). ***Sexual morph***: Undetermined.

**Figure 2.**
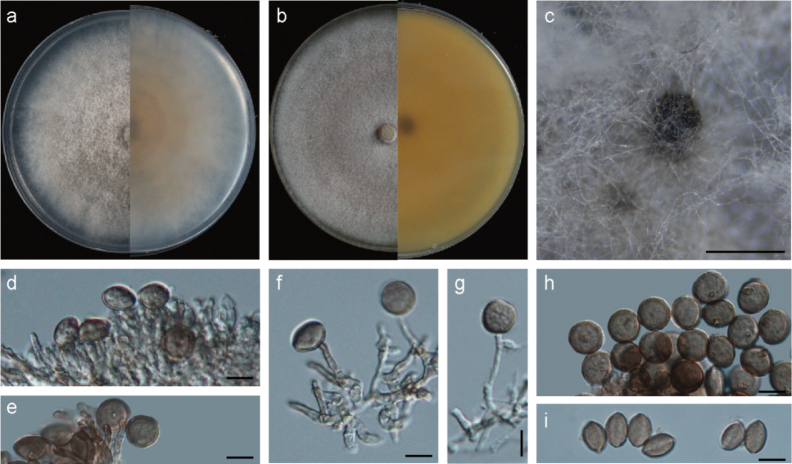
*Apiosporagongcheniae* (YNE00465, ex-type culture) **a** colonies after 7 d at 25 °C on PDA**b** colonies after 7 d at 25 °C on MEA**c** conidiomata on MEA**d-g** conidiogenous cells giving rise to conidia **h–i** conidia with pale germ slit. Scale bars: 500 μm (**e**); 10 μm (**f–k**).

##### Culture characteristics.

On PDA, colonies flat, cottony, dense, margin circular, greyish, reverse light orange, covering the 90 mm plate after 7 days at 25 °C. On MEA, colonies dusty pink, dense, covering the 90 mm plate after 7 days at 25 °C. Conidiomata black, globose, abundant, attach to surface of substrate, forming on PDA and MEA after 7–10 days.

##### Additional specimens examined.

China, Yunnan Province: Xishuangbanna, Naban River Watershed National Nature Reserve, 22°04'N, 100°32'E, on the stems of Oryzameyerianasubsp.granulata, Aug 2015, J.J. Chen, YNE00565.

##### Note.

Phylogenetic analyses confirmed that *A.gongcheniae* formed an independent clade, exhibiting a close evolutionary relationship with *A.garethjonesii*, *A.neogarethjonesii* and *A.subrosea*. Based on a BLASTN search of the GenBank database, it was found that *A.paragongcheniae* shares high similarities with the following strains: *A.garethjonesii* strain HKAS 96289 (93.76% in ITS, 99.81% in LSU), strain GZCC 20-0115 (93.76% in ITS, 99.24% in LSU, 94.06% in *tef1*), strain SICAUCC 22-0027 (93.76% in ITS, 99.81% in LSU, 94.51% in *tub2*), strain SICAUCC 22-0028 (93.76% in ITS, 99.81% in LSU, 93.63% in *tub2*); *A.subrosea* strain CGMCC 3.18337 (96.94% in ITS, 99.42% in LSU, 93.47% in *tef1*, 91.87% in *tub2*), strain LC7291 (90.09% in ITS, 99.41% in LSU, 93.47% in *tef1*, 91.87% in *tub2*); and *A.neogarethjonesii* strain HKAS 102408 (92.86% in ITS, 99.82% in LSU). The *tef1* and *tub2* sequence data are currently unavailable for *A.neogarethjonesii* to compare with *A.gongcheniae*.

As a synopsis of the morphological characteristics presented in Table 2, *A.gongcheniae* differs from *A.garethjonesii* and *A.neogarethjonesii* in having smaller conidia (8.0–17.0 × 6.8–16.1 μm, mean = 13.6 × 11.6 μm) compared to *A.garethjonesii* (surface view: 16–19 µm diam, side view: 17–22 µm diam) and *A.neogarethjonesii* (20–35 × 15–30 µm, mean = 28.5 × 25.6 µm). Additionally, *A.gongcheniae* exhibits shorter conidiogenous cells (3.5–9.4 × 1.9–5.2 μm, mean = 5.6 × 3.1 μm) in contrast to *A.garethjonesii* (6–19 × 3–5 µm, mean = 11 × 4 µm) and *A.neogarethjonesii* (10–48 × 4–5.5 µm, mean = 35.4 × 4.3 µm). While *A.gongcheniae* shares a similar size range for conidia and conidiogenous cells with *A.subrosea*, it is distinguished by *A.gongcheniae* having conidia featuring a central basal scar and cylindrical conidiogenous cells with tiny denticles. Based on molecular and morphological evidence, we propose *A.gongcheniae* as a new species.

**Table 2.** Synopsis of morphological characteristics of related *Apiospora* species. Notes: ND = Not determined.

Strains	*Apiosporagarethjonesii* (D.Q. Dai & H.B. Jiang) Pintos & P. Alvarado (2021)	*A.neogarethjonesii* (D.Q. Dai & K.D. Hyde) Pintos & P. Alvarado (2021)	*A.subrosea* (M. Wang & L. Cai) Pintos & P. Alvarado (2021)	*A.neobambusae* Pintos & P. Alvarado (2021) (=*Arthriniumbambusae* M. Wang & L. Cai (2018))	*A.gongcheniae*	*A.paragongcheniae*	*A.neogongcheniae*
**Host / Substrate**	Dead culms of bamboo	Dead culms of bamboo	Leaves of bamboo	Leaves of bamboo	Stems of Oryzameyerianasubsp.granulata	Stems of unidentified Poaceae plant	Stems of unidentified Poaceae plant
**Known lifestyle**	Saprobe	Saprobe	Endophyte	Endophyte	Endophyte	Endophyte	Endophyte
**Asci**	125–154 × 35–42 μm (x– = 139 × 38 μm, n = 20), 8-spored	95–125 × 20–25 μm (x– = 97.6 × 21.3 μm, n = 20), 8-spored	ND	ND	ND	ND	ND
**Ascospore**s	30–42 × 11–16 μm (x– = 39 × 13 μm, n = 20), 2-seriate, 1-septate, ellipsoidal	25–30 × 9.5–11 μm (x– = 29.1 × 10.3 μm, n = 20), 2-seriate, overlapping, 1-septate, ellipsoidal, 3–10 µm wide	ND	ND	ND	ND	ND
**Conidiomata**	Black, with hair-like setae	Black, ellipsoid to irregular, coriaceous	Black, irregular	Black, irregular	Black, globose, abundant, attach to the surface of the substrate	Black, globose to irregular shape, sparse, semi-immersed in the substrate	ND
**Conidiophores**	Reduced to conidiogenous cells	4.5–6 × 3.5–4.5 µm (x– = 5.4 × 4.3 µm, n = 20), cylindrical, aseptate	Hyaline to pale brown, smooth, erect or ascending, simple, flexuous, subcylindrical, clustered in groups, aggregated in brown sporodochia, up to 20 µm long, 2–4.5 µm width	Reduced to conidiogenous cells	Reduced to conidiogenous cells	Hyaline, erect, basauxic, doliiform, subspherical to barrel-shaped, aggregated in clusters on pale brown sporodochia, sometimes reduced to conidiogenous cells, 12.2–35.1 × 2.1–8.8 μm (x– = 24.5 × 4.3 μm, n = 30)	ND
**Conidiogenous cells**	Hyaline to pale brown, smooth, ampulliform, aggregated in black sporodochia, (5−) 6–19 (−20) µm × (2−) 3–5 (−7) µm (x– = 11 µm × 4 µm, n = 20)	Basauxic, cylindrical, discrete, smooth-walled, 10–48 × 4–5.5 µm (x– = 35.4 × 4.3 µm, n = 20)	Pale brown, smooth, doliiform to subcylindrical, 3.0–6.5 × 2.0–5.0 µm (x– = 4.7 ± 1.2 × 3.7 ± 0.9, n = 30)	Hyaline to pale brown, erect, aggregated in clusters on hyphae, smooth, doliiform to ampulliform, or lageni-form, 4.0–12.0 × 3.0–7.0 µm (x– = 6.6 ± 1.8 × 4.8 ± 0.9, n = 30)	Hyaline to pale brown, erect, verrucose, cylindrical with tiny denticles, clustered in groups, sometimes aggregated in clusters on hyphae or sporodochia, 3.5–9.4 × 1.9–5.2 μm (x– = 5.6 × 3.1 μm, n = 30)	Hyaline, ampulliform, doliiform to clavate, verrucose, 5.0–13.1 × 2.1–6.0 μm (x– = 8.2 × 3.9 μm, n = 30)	ND
**Conidia**	(14–)16–19 (–20) µm diam, brown, smooth, granular, globose to subglobose in surface view, and (16−) 17–22 (−23) µm diam, with pale equatorial slit in side view	Dark brown, globose to subglobose, smooth-walled, with a truncate basal scar, 20–35 × 15–30 µm (x– = 28.5 × 25.6 µm, n = 20)	Pale brown to dark brown, smooth, globose to subglobose or ellipsoidal, 12.0–17.5 × 9.0–16.0 µm (x– = 14.9 ± 1.4 × 11.8 ± 1.8, n = 50)	Olivaceous to brown, smooth to finely roughened, subglobose to ellipsoid, 11.5–15.5 × 7.0–14.0 µm (x– = 13.2 ± 0.8 × 11.4 ± 1.2, n = 50)	Pale brown to dark brown, smooth, granular, globose to subglobose in surface view, lenticular to side view with a pale longitudinal germ slit, with obvious central basal scar, 8.0–17.0 × 6.8–16.1 μm (x– = 13.6 × 11.6 μm, n = 50)	Pale brown to dark brown, smooth to granular, subglobose to oval, occasionally swollen into pyriform to reniform, with a pale longitudinal germ slit in side view, 8.2–18.7 × 6.4–13.4 μm (x– = 12.4 × 10.0 μm, n = 50)	ND
**References**	(Dai et al. 2016; Feng et al. 2021)	(Hyde et al. 2020)	(Wang et al. 2018)	(Wang et al. 2018)	This study	This study	This study

#### ﻿ Apiospora paragongcheniae

Taxon classificationFungiXylarialesApiosporaceae

C. L. Zhang sp. nov.

4B35590E-B30E-5BC6-8608-CEE8CD1E6423

Fungal Names: FN 571886

Fig. 3

##### Etymology.

Named after its phylogenetic close related to *A.gongcheniae*.

##### Type.

China, Yunnan Province: Xishuangbanna, Naban River Watershed National Nature Reserve, 22°04'N, 100°32'E, on the stems of unidentified Poaceae plant, Sep 2016, J.J. Chen, YNE00992 (Holotype GDMCC 3.1046, stored in a metabolically inactive state); ex-type culture YNE00992.

##### Description.

***Asexual morph***: Hyphae hyaline, branched, septate, smooth, 1.1–2.2 μm diameter (mean = 1.6 μm, n = 30). Conidiophores hyaline, erect, basauxic, doliiform, subspherical to barrel-shaped, aggregated in clusters on pale brown sporodochia, sometimes reduced to conidiogenous cells, 12.2–35.1 × 2.1–8.8 μm (mean = 24.5 × 4.3 μm, n = 30). Conidiogenous cells hyaline, ampulliform, doliiform to clavate, verrucose, 5.0–13.1 × 2.1–6.0 μm (mean = 8.2 × 3.9 μm, n = 30). Conidia pale brown to dark brown, smooth to granular, subglobose to oval, occasionally swollen into pyriform to reniform, with a pale longitudinal germ slit in side view, 8.2–18.7 × 6.4–13.4 μm (mean = 12.4 × 10.0 μm, n = 50). ***Sexual morph***: Undetermined.

##### Culture characteristics.

On PDA, colonies flat, rounded, initially white, becoming yellowish-white, with sparse aerial mycelia, mycelium partly immersed in the medium, covering the 90 mm plate after 6 days at 25 °C. On MEA, colonies white, more abundant aerial mycelia, covering the 90 mm plate after 6 days at 25 °C. Conidiomata black, globose to irregular shape, sparse, solitary, semi-immersed in the substrate, observed on MEA after 21–30 days.

##### Additional specimens examined.

China, Yunnan Province: Xishuangbanna, Naban River Watershed National Nature Reserve, 21°10'N, 99°55'E, on the stems of unidentified Poaceae plant, Oct 2018, X.X. Feng, YNE001259.

##### Note.

Phylogenetic analyses confirmed that *A.paragongcheniae* formed an independent clade, exhibiting a close evolutionary relationship with *A.subrosea*, *A.neobambusae* and *A.neogarethjonesii*. Based on a BLASTN search of the GenBank database, it was found that *A.paragongcheniae* shares high similarities to the following strains: *A.subrosea* strain CGMCC 3.18337 (98.05% in ITS, 99.23% in LSU, 95.93% in *tef1*, 93.63% in *tub2*), strain LC7291 (98.05% in ITS, 99.22% in LSU, 95.93% in *tef1*, 93.63% in *tub2*); *A.neobambusae* strain CGMCC 3.18335 (98.05% in ITS, 100% in LSU, 97.13% in *tef1*, 93.48% in *tub2*), strain LC7107 (98.03% in ITS, 100% in LSU, 94.44% in *tef1*, 93.48% in *tub2*), strain LC7124 (98.05% in ITS, 100% in LSU, 96.82% in *tef1*, 93.47% in *tub2*); and *A.neogarethjonesii* strain HKAS 102408 (95.43% in ITS, 99.63% in LSU). The *tef1* and *tub2* sequence data are currently unavailable for *A.neogarethjonesii* to compare with *A.paragongcheniae*.

As a synopsis of morphological characteristics presented in Table 2, *A.paragongcheniae* distinguishes itself from *A.neobambusae*, *A.neogarethjonesii*, and *A.subrosea* in the shapes and sizes of its conidia. The conidia of *A.paragongcheniae* range from subglobose to oval, occasionally swollen into pyriform to reniform shapes, measuring 8.2–18.7 × 6.4–13.4 μm. This contrasts with *A.neobambusae* (subglobose to ellipsoid, 11.5–15.5 × 7.0–14.0 µm), *A.neogarethjonesii* (globose to subglobose, 20–35 × 15–30 µm), and *A.subrosea* (globose to subglobose or ellipsoidal, 12.0–17.5 × 9.0–16.0 µm). Furthermore, *A.paragongcheniae* exhibits elongated conidiogenous cells (5.0–13.1 × 2.1–6.0 μm, mean = 8.2 × 3.9 μm) compared to *A.neobambusae* (4.0–12.0 × 3.0–7.0 µm, mean = 6.6 × 4.8 μm) and *A.subrosea* (3.0–6.5 × 2.0–5.0 µm, mean = 4.7 × 3.7 μm). Additionally, *A.paragongcheniae* exhibits shorter conidiogenous cells (5.0–13.1 × 2.1–6.0 μm) compared to *A.neogarethjonesii* (10–48 × 4–5.5 µm). Moreover, these species differ in the morphology of their conidiophores. *A.paragongcheniae* displays hyaline, basauxic, doliiform, subspherical to barrel-shaped conidiophores, whereas *A.neogarethjonesii* has shorter conidiophores, and *A.subrosea* has hyaline to pale brown, simple, subcylindrical conidiophores. Notably, the conidiophores of *A.neobambusae* have reduced to conidiogenous cells.

**Figure 3.**
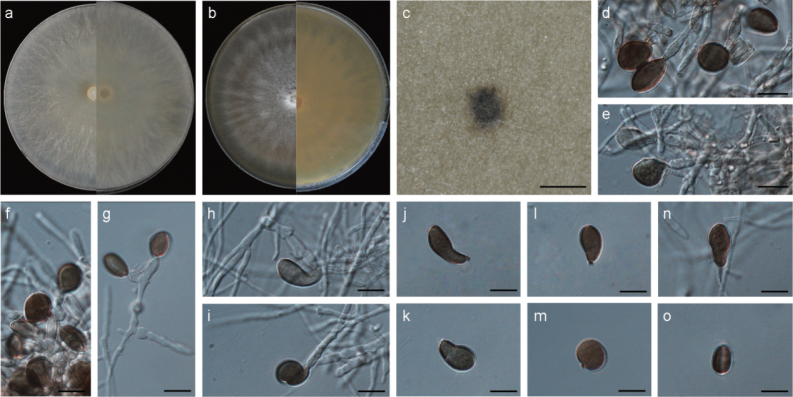
*Apiosporaparagongcheniae* (YNE00992, ex-type culture) **a** colonies after 7 d at 25 °C on PDA**b** colonies after 6 d at 25 °C on MEA**c** conidioma on MEA**d–i** conidiogenous cells giving rise to conidia **j–o** conidia. Scale bars: 500 μm (**c**); 10 μm (**d–o**).

#### ﻿ Apiospora neogongcheniae

Taxon classificationFungiXylarialesApiosporaceae

C. L. Zhang sp. nov.

658C79C8-D19C-517C-BED6-D851C9B7EDF9

Fungal Names: FN 571887

Fig. 4

##### Etymology.

Named after its phylogenetic close related to *A.gongcheniae*.

##### Type.

China, Yunnan Province: Xishuangbanna, Naban River Watershed National Nature Reserve, 21°10'N, 99°55'E, on the stems of unidentified Poaceae plant, Oct 2018, X.X. Feng, YNE01248 (holotype GDMCC 3.1047, stored in a metabolically inactive state); ex-type culture YNE01248.

##### Description.

***Asexual morph***: Hyphae hyaline, branched, septate, smooth, 1.0–2.5 μm diameter (mean = 1.5 μm, n = 30). Conidia not observed. Chlamydospores single, terminal, globose, rare. ***Sexual morph***: Undetermined.

##### Culture characteristics.

On PDA, colonies flat, rounded, initially white, becoming yellowish-white, cottony, with moderate aerial mycelia, covering the 90 mm plate after 7 days at 25 °C. On MEA, colonies white, dense aerial mycelia, forming multiple circles around the center, covering the 90 mm plate after 7 days at 25 °C. Conidiomata were not observed.

##### Additional specimens examined.

China, Yunnan Province: Xishuangbanna, Naban River Watershed National Nature Reserve, 21°10'N, 99°55'E, on the stems of unidentified Poaceae plant, Oct 2018, X.X. Feng, YNE001260.

##### Note.

Phylogenetic analyses confirmed that *A.neogongcheniae* formed an independent clade, exhibiting a close evolutionary relationship with *A.garethjonesii*, *A.neogarethjonesii* and *A.subrosea*. Based on a BLASTN search of the GenBank database, it was found that *A.neogongcheniae* shares high similarities with the following strains: *A.garethjonesii* strain HKAS 96289 (94.88% in ITS, 100% in LSU), strain GZCC 20-0115 (94.88% in ITS, 99.41% in LSU, 96.67% in *tef1*), strain SICAUCC 22-0027 (94.88% in ITS, 100% in LSU, 96.69% in *tub2*), strain SICAUCC 22-0028 (94.88% in ITS, 100% in LSU; 96.79% in *tub2*); *A.subrosea* strain CGMCC 3.18337 (98.35% in ITS, 99.80% in LSU, 94.61% in *tef1*, 94.99% in *tub2*), strain LC7291 (91.41% in ITS, 99.80% in LSU, 94.38% in *tef1*, 94.99% in *tub2*); and *A.neogarethjonesii* strain HKAS 102408 (93.97% in ITS, 100% in LSU). The *tef1* and *tub2* sequence data are currently unavailable for *A.neogarethjonesii* to compare with *A.neogongcheniae*.

Due to the absence of sexual and asexual sporulation characters in *A.neogongcheniae*, a comparison of its culture characteristics with those of *A.garethjonesii*, *A.neogarethjonesii* and *A.subrosea* was conducted. On PDA, *A.neogongcheniae* exhibits a yellowish-white surface and reverse color, whereas *A.garethjonesii* displays a white surface with a reddish reverse, *A.neogarethjonesii* shows a white to black surface coloration, and *A.subrosea* presents a light pink surface with a peach-puff reverse. Phylogenetically, *A.neogongcheniae* strains YNE01248 and YNE01260 form a distinct branch with 99% MLBP and 0.95 BIPP. Therefore, we propose *A.neogongcheniae* as a novel species.

**Figure 4.**
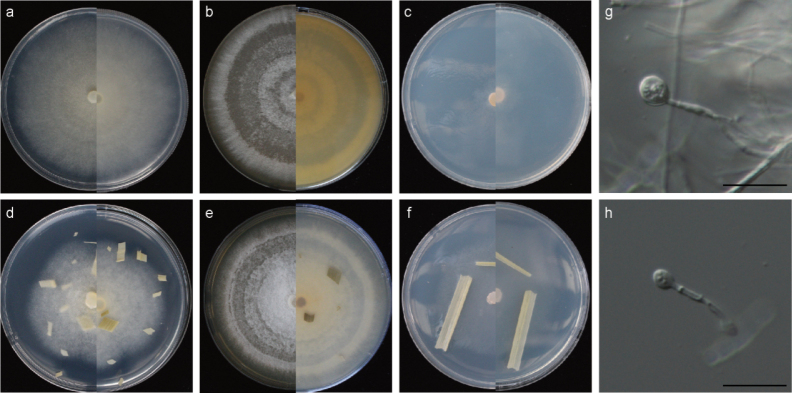
*Apiosporaneogongcheniae* (YNE01248, ex-type culture) **a** colonies after 7 d at 25 °C on PDA**b** colonies after 7 d at 25 °C on MEA**c** colonies after 7 d at 25 °C on SNA **d** colonies after 7 d at 25 °C on PDA with rice leaves **e** colonies after 7 d at 25 °C on MEA with rice leaves **f** colonies after 7 d at 25 °C on SNA with rice leaves **g–h** chlamydospores. Scale bars: 20 μm.

## ﻿Discussion

In the present study, three new species of endophytic *Apiospora* were examined: *A.gongcheniae*, *A.paragongcheniae*, and *A.neogongcheniae*, all of them isolated from the stems of Poaceae plants in Yunnan province of China. According to morphological and molecular identification, the taxonomic position of the three new species was verified.

The generic circumscription of *Apiospora* was primarily defined through phylogenetic analysis, given the limited morphological characteristics of *Apiospora* and *Arthrinium*. The results of a multi-locus phylogenetic analysis in this study, utilizing a combined dataset of ITS, LSU, *tef1*, and *tub2* sequences, supported the previous classification that *Apiospora* and *Arthrinium* are distinct lineages rather than synonyms (Pintos and Alvarado 2021). Unlike the six major clades identified in a previous study (Pintos and Alvarado 2022), the current study revealed twelve major clades with robust support through the phylogenetic analysis of 114 *Apiospora* species, including all known species with available sequences. *Apiosporaminutispora* (Das et al. 2020) and *Apiosporamarianiae* AP18219 (Pintos and Alvarado 2022) were not classified within these twelve major clades due to their representation by a single record. The delineation of most *Apiospora* species into major clades remained consistent across both studies. Notably, *A.garethjonesii*, *A.neogarethjonesii*, *A.neobambusae*, *A.mytilomorpha*, *A.subrosea*, and *A.setostroma* clustered together in a strongly supported major clade H, aligning with findings from previous studies (Crous et al. 2021; Monkai et al. 2022; Pintos and Alvarado 2022; Liao et al. 2023; Liu et al. 2024). Within this major clade, three distinct clades representing three new species were identified (Fig. 1). *A.gongcheniae* is distinguished from *A.garethjonesii* by 34/545 nucleotides in the ITS sequences, from *A.neogarethjonesii* by 39/546, and from *A.subrosea* by 13/425. *A.paragongcheniae* is distinguished from *A.subrosea* by 10/512, from *A.neobambusae* by 10/512, and from *A.neogarethjonesii* by 24/525 nucleotides in the ITS sequences. *A.neogongcheniae* is distinguished from *A.garethjonesii* by 28/547, from *A.neogarethjonesii* by 34/547, and from *A.subrosea* by 7/425 nucleotides in the ITS sequences.

*Apiospora* exhibits ecological diversity, as evidenced by its wide host ranges. Most reported *Apiospora* species show a host preference within the Poaceae family, as noted by Monkai et al. (Monkai et al. 2022). Our new species were also found growing on plant hosts of the Poaceae family. Specifically, *A.gongcheniae* was discovered on the stems of Oryzameyerianasubsp.granulata, a member of the plant family Poaceae. The other two new species, *A.paragongcheniae* and *A.neogongcheniae*, were found on the stems of unidentified Poaceae plants. Their close relatives, *A.garethjonesii*, *A.neogarethjonesii*, *A.neobambusae*, and *A.subrosea*, were found on bamboo plants. Most *Apiospora* species exhibit saprobic and endophytic lifestyles, which are likely associated with the prevalence of *Apiospora* (Liao et al. 2023). Our new species occurred as endophytic fungi. Further investigation into endophytic *Apiospora* species will significantly enhance the diversity within the *Apiospora* genus.

Morphological characteristics, including asexual and sexual structures, serve as a fundamental basis for fungal systematics and phylogenetic studies, playing a vital role in the comprehensive examination of fungi. However, many endophytes do not form distinct asexual and sexual structures, as observed in *A.neogongcheniae* in this study, posing challenges in determining their taxonomic status based on morphological features. Recent advances in fungal taxonomy and phylogeny have provided new insights into many species with limited morphological features. Future taxonomic efforts necessitate the integration of morphological traits with molecular evidence to elucidate the natural and stable phylogenetic relationships among *Apiospora* species and their related *Arthrinium* species.

## Supplementary Material

XML Treatment for Apiospora gongcheniae

XML Treatment for Apiospora paragongcheniae

XML Treatment for Apiospora neogongcheniae

## ﻿Additional information

### Author ORCIDs

Xiao-Ni Yan https://orcid.org/0009-0009-9984-3617

Chu-Long Zhang https://orcid.org/0000-0001-5180-0348

### Data availability

All of the data that support the findings of this study are available in the main text.
